# Supplementary material for: C25-modified rifamycin derivatives with improved activity against Mycobacterium abscessus
Source: PNAS Nexus. 2022 Aug 9;1(4):pgac130. doi: 10.1093/pnasnexus/pgac130 (PMC9802118; doi:10.1093/pnasnexus/pgac130)
Supplement: pgac130_Supplemental_File [file pgac130_supplemental_file.docx]

Supplementary Materials for

C25-modified rifamycin derivatives with improved activity against *Mycobacterium abscessus*

Laura Paulowski^1,†^, Katherine S. H. Beckham^2,†^, Matt D. Johansen^3,†^, Laura Berneking^4,†^, Nhi Van^5^, Yonatan Degefu^5^, Sonja Staack^2^, Flor Vasquez Sotomayor^1,4^, Lucia Asar^4^, Holger Rohde^4^, Bree B. Aldridge^5^, Martin Aepfelbacher^4^, Annabel Parret^2,7^, Matthias Wilmanns^2,8^, Laurent Kremer^3,9^, Keith Combrink^10,11^, Florian P. Maurer^1,4,12,^**^*^**

*Corresponding author.

Email: fmaurer@fz-borstel.de

**This PDF file includes:**

Supplementary

Figure S1

Tables S1 to S3

Figure S1


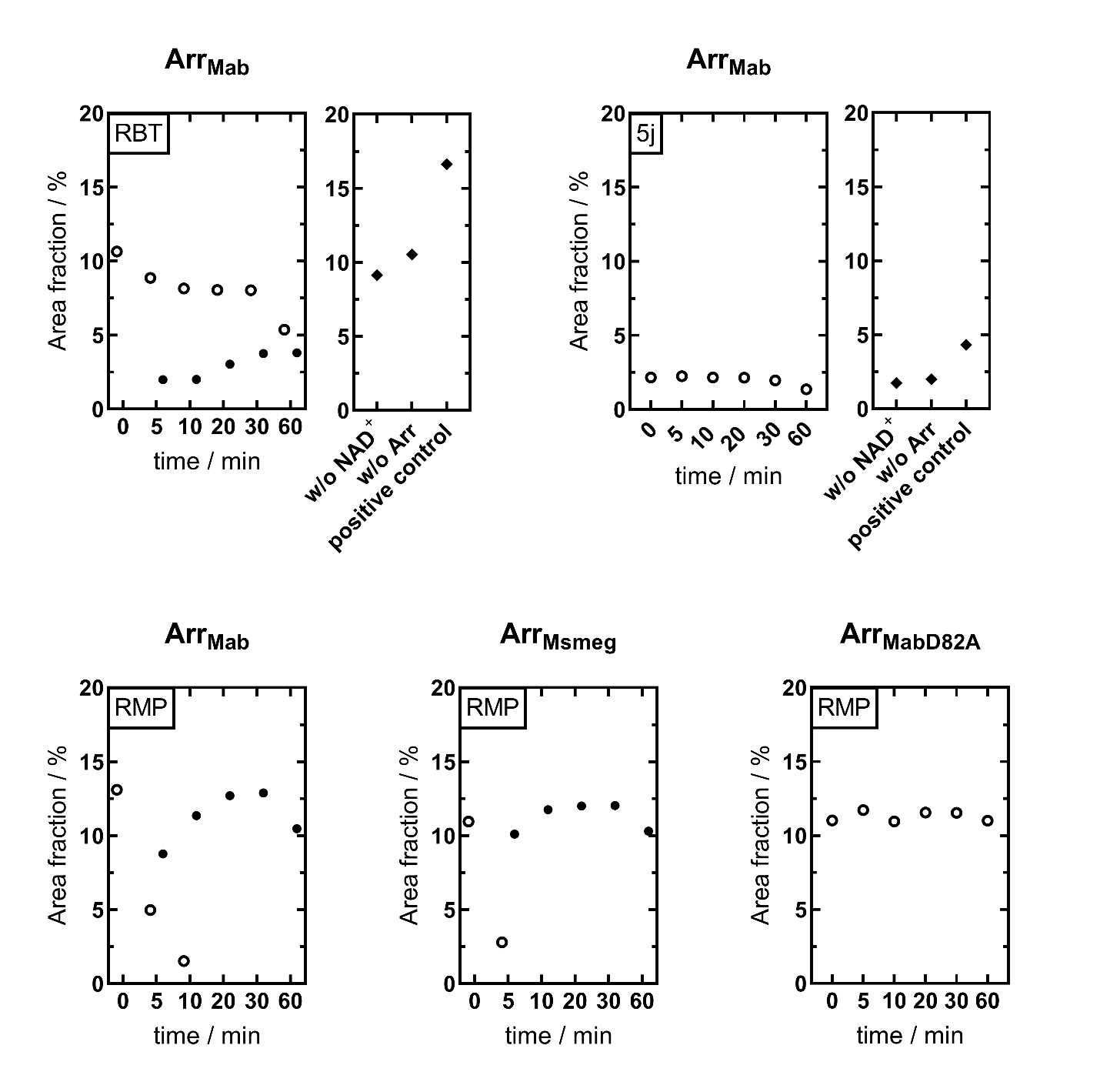


Figure S1: Densitometric analysis of reverse phase thin layer chromatography (rpTLC) of ADP-ribosylation reactions. The greyscale intensities of the rpTLC bands were measured using ImageJ and the relative areas under each peak of the corresponding ImageJ histograms were plotted as dots for each time point. Primary bands are depicted by white (○) and secondary bands by black (●) circles. Arr ADP-ribosyltransferases were expressed and purified from *M. abscessus* (Arr_Mab_) or *M. smegmatis* (Arr_Msm_). Reactions without NAD^+^ (w/o NAD+) or without enzymes (w/o Arr) were added as negative controls. RMP was used as positive control. Control data sets are illustrated for RBT and 5j.

Table S1.

Compound designations and reference for synthesis methodology.

| Compound | Designation in Combrink *et al.* 2019 | Substituent R1 | Synthesis | NMR | MS |
| --- | --- | --- | --- | --- | --- |
| 5f | 2a | 1-methoxy-4-methylbenzene | [34,41] | [34] | [34] |
| 5g | 2b | *N,N*,4-trimethylaniline | [34,41] | [34] | [34] |
| 5j | 2g | 4-(benzylamino)-1-piperidine | [34,41] | [34] | [34] |
| 5k | 2d | 5-ethyl-3-(2-methoxy)phenylisoxazole | [34,41] | [34] | [34] |
| 5l | 2e | 2-(5-ethyl-3-isoxazolyl)pyridine | [34,41] | [34] | [34] |
| 5n | 2f | Propyldiisopropylamine | [34,41] | [34] | [34] |

Table S2.

Plasmids and primers used to clone Arr_Mab_, Arr_Msm_, and ArrD82A_Mab_.

| Plasmid | Insert | Primers (Forward, F; Reverse, R) |
| --- | --- | --- |
| pCoofy1-MabsArr | MAB_0591 | F-TCTGTTCCAGGGGCCCATGGTGACGATGCCCAACTTTTTCGAGGC |
|  |  | R-GTGGTGCTCGAGTGCGGCCGCTCAGTCATAGATGACCGCGTTTCC |
| pCoofy1-MsmegArr | MSMEG_1221 | F-TCTGTTCCAGGGGCCCATGGGCGTGGCGAATCCGCCGAAACCGTT |
|  |  | R-GTGGTGCTCGAGTGCGGCCGCCTAGTCATAGATGACCGCCAGCC |
| pCoofy1-MabsArrD82A | MAB_0591 D82A | F-CTGGAAGACGCCCCCAACGTG |
|  |  | R-GTGGAACCGGAAGGCACC |

**Table S3.**

**Mean MIC values of RMP, RBT, and rifamycin derivatives against *M. abscessus* ATCC 19977 and clinical NTM isolates.** MIC values are given in μg/mL with the corresponding micromolar values in parentheses. MIC values were obtained for nonduplicate clinical isolates of *M. abscessus* subsp. *abscessus* (n=9), *M. abscessus* subsp. *bolletii* (n=7), *M. abscessus* subsp. *massiliense* (n=8), *M. chelonae* (n=8), and *M. fortuitum* (n=8).

| compound | *M. abscessus*  ATCC 19977 | clinical isolates | | |
| --- | --- | --- | --- | --- |
|  |  | ***M. abscessus*** | ***M. chelonae*** | ***M. fortuitum*** |
| RMP | 128 (155.54) | 128 (155.54) | 128 (155.54) | 128 (155.54) |
| RBT | 4 (4.72) | 3.58 (4.23) | 3.25 (3.84) | 2.5 (2.95) |
| 5d | 16 (18.76) | 12.17 (14.27) | 13.38 (15.68) | 1.19 (1.39) |
| 5f | 128 (141.91) | 45.74 (50.71) | 97.25 (107.82) | 1.38 (1.52) |
| 5j | 2 (2.1) | 0.24 (0.26) | 0.13 (0.13) | 0.71 (0.74) |
| 5k | 32 (33.02) | 49.39 (50.97) | 33.38 (34.44) | 1.03 (1.07) |
| 5l | 8 (8.51) | 6.65 (7.08) | 11.13 (11.84) | 2.58 (2.74) |
| 5n | 2 (2.2) | 0.82 (0.89) | 0.83 (0.91) | 5.23 (5.76) |
